# Maximising harm reduction in early specialty training for general practice: validation of a safety checklist

**DOI:** 10.1186/1471-2296-13-62

**Published:** 2012-06-21

**Authors:** Paul Bowie, John McKay, Moya Kelly

**Affiliations:** 1 Department of Postgraduate General Practice Education, NHS Education for Scotland, 2 Central Quay, Glasgow, UK, G3 8BW

## Abstract

**Background:**

Making health care safer is a key policy priority worldwide. In specialty training, medical educators may unintentionally impact on patient safety e.g. through failures of supervision; providing limited feedback on performance; and letting poorly developed behaviours continue unchecked. Doctors-in-training are also known to be susceptible to medical error. Ensuring that all essential educational issues are addressed during training is problematic given the scale of the tasks to be undertaken. Human error and the reliability of local systems may increase the risk of safety-critical topics being inadequately covered. However adherence to a checklist reminder may improve the reliability of task delivery and maximise harm reduction. We aimed to prioritise the most safety-critical issues to be addressed in the first 12-weeks of specialty training in the general practice environment and validate a related checklist reminder.

**Methods:**

We used mixed methods with different groups of GP educators (n = 127) and specialty trainees (n = 9) in two Scottish regions to prioritise, develop and validate checklist content. Generation and refinement of checklist themes and items were undertaken on an iterative basis using a range of methods including small group work in dedicated workshops; a modified-Delphi process; and telephone interviews. The relevance of potential checklist items was rated using a 4-point scale content validity index to inform final inclusion.

**Results:**

14 themes (e.g. prescribing safely; dealing with medical emergency; implications of poor record keeping; and effective & safe communication) and 47 related items (e.g. how to safety-net face-to-face or over the telephone; knowledge of practice systems for results handling; recognition of harm in children) were judged to be essential safety-critical educational issues to be covered. The mean content validity index ratio was 0.98.

**Conclusion:**

A checklist was developed and validated for educational supervisors to assist in the reliable delivery of safety-critical educational issues in the opening 12-week period of training, and aligned with national curriculum competencies. The tool can also be adapted for use as a self-assessment instrument by trainees to guide patient safety-related learning needs. Dissemination and implementation of the checklist and self-rating scale are proceeding on a national, voluntary basis with plans to evaluate its feasibility and educational impact.

## Background

Patients worldwide are unintentionally but avoidably harmed as a result of their interactions with health care [1]. A global campaign to highlight the patient safety problem and recommend potential solutions is being led by the World Health Organisation (WHO) [2]. In the United Kingdom (UK), making patient care safer has been an explicit policy priority in the National Health Service (NHS) for over a decade [3]. Until recently the predominant safety improvement focus was on acute hospital settings where there is well-established evidence of the scale and consequences of adverse health care events [4]. In UK general medical practice comparable research and knowledge of the nature and impact of avoidable harm is growing, but is methodologically limited compared with secondary care [5,6]. However, the emerging evidence from a range of international sources suggests that the safety of general practice may be compromised in a significant minority of cases [7-9]. Specialty trainees are known to be involved in a proportion of these incidents largely because of a range of systems, knowledge, cognitive, training and behaviour based reasons.

In the NHS, a raft of interventions designed to make patient care safer are being implemented or are under development. For example: multi-centre collaborative programmes of safety improvement that aim to reduce harm in specific areas of acute hospital care and, more recently, primary care are currently underway [10]; a high-profile and long-running campaign to minimize healthcare acquired infections is ongoing [11]; while a concerted attempt to improve the reporting and learning from patient safety incidents has also taken place [12].

From an educational perspective, it is now recognised that there is a need for patient safety education to be more explicitly integrated and prominently positioned within existing undergraduate and postgraduate training curricula for all health care professions [13]. The UK Royal College of General Practitioners (RCGP) - which has responsibility for the content of the specialty training curriculum - has responded by developing a curriculum statement on ‘patient safety’ [14] and defining specific learning objectives (Table 1). However, it is left to individual GP educational supervisors to determine how the RCGP curriculum is best delivered and the related learning needs of trainees are identified and acted upon during the training period. The role of the postgraduate deaneries in this regard is to verify that the evidence provided by training practices to support the delivery of the patient safety element of the curriculum is of an adequate standard.

**Table 1:** RCGP Curriculum Learning Outcomes (with examples) related to Patient Safety

**Learning Outcome**	**Description and Example of Outcome**
**1.**	**Primary Care Management**
	(e.g. Contribute to the regular significant event audit (SEA) meetings and observe the benefits of a multidisciplinary team)
**2.**	**Person-Centred Care**
	(e.g. Communicate openly, listen and take patients’ concerns seriously. Consider patient issues when reflecting on consultation experiences)
**3.**	**Specific Problem-Solving Skills**
	(e.g. Demonstrate an awareness of the limitations of your own skills in risk management and illustrate that youunderstand when the skills of colleagues trained more extensively in risk management should be called upon)
**4.**	**A Comprehensive Approach**
	(e.g. Describe the risks to patient safety by considering an illness pathway/journey in which a variety of healthcare professionals have been involved)
**5.**	**Community Orientation**
	(e.g. Describe how patient groups may be put at increased risk of mishap by virtue of their particular characteristics, such as language, literacy, culture and health beliefs)
**6.**	**A Holistic Approach**
	(e.g. Describe how the lessons of patient safety can be applied prospectively to doctor–patient interactions, especially through the identification and discussion of risk).
**7.**	**Contextual Aspects**
	(e.g. Describe the impact of the working environment on the care the doctor provides and the likelihood of adverse incidents as a result of this)
**8.**	**Attitudinal Aspects**
	(e.g. Help to shape an organisational culture that prioritises safety and quality through openness, honesty, shared learning and continual incremental improvement)
**9.**	**Scientific Aspects**
	(e.g. Describe the basic principles of risk assessment)

*UK GP Specialty Trainees are required to spend 18 months in a GP setting as part of a 3 or 4 year programme. The teaching required is governed by the RCGP curriculum and one area that is increasingly being highlighted is UK general practice is patient safety.

Ensuring that all essential educational issues are identified, prioritised and satisfactorily covered during training is not straightforward given the complexity of the tasks to be undertaken and the high volume of topics to be addressed. Within the framework of the RCGP curriculum, GP educational supervisors currently guide the activities undertaken by the trainee using a combination of locally developed induction packs, nationally promoted learning interventions and assessments, and professional experience in the workplace to match across to RCGP curriculum competencies (Table 2). However, it is currently unclear how the patient safety-related learning needs of trainees are specifically addressed when in the training environment. Given the lack of standardized guidance on how and what specific learning issues are to be covered, it is possible there is variation in GP educational supervisors’ interpretation and delivery of this safety-critical element of the curriculum at the ‘sharp end’ of frontline educational practice.

**Table 2:** A list of the 12 RCGP Curriculum Competencies with descriptions (assessment scale: insufficient evidence; needs further development; competent; and excellent)

**Competency No.**	**Description of Competency**
**1.**	**Communication and consultation skills**
	(This competency is about communication with patients, and the use of recognised consultation techniques)
**2.**	**Practising holistically**
	(This competency is about the ability of the doctor to operate in physical, psychological, socio-economic and cultural dimensions, taking into account feelings as well as thoughts)
**3.**	**Data gathering and interpretation**
	(This competency is about the gathering and use of data for clinical judgement, the choice of examination and investigations and their interpretation)
**4.**	**Making a diagnosis/making decisions**
	(This competency is about a conscious, structured approach to decision-making)
**5.**	**Clinical management**
	(This competency is about the recognition and management of common medical conditions in primary care)
**6.**	**Managing medical complexity**
	(This competency is about aspects of care beyond managing straightforward problems, including the management of co-morbidity, uncertainty and risk, and the approach to health rather than just illness).
**7.**	**Primary care administration and information management & technology**
	(This competency is about the appropriate use of primary care administration systems, effective record keeping and information technology for the benefit of patient care)
**8.**	**Working with colleagues and in teams**
	(This competency is working effectively with other professionals to ensure patient care, including the sharing of information with colleagues)
**9.**	**Community orientation**
	(This competency is about the management of the health and social care of the practice population and local community)
**10.**	**Maintaining performance, learning and teaching**
	(This competency is about maintaining the performance and effective continuing professional development of oneself and others)
** 11.**	**Maintaining an ethical approach to practise**
	**(**This competency is about practising ethically with integrity and a respect for diversity)
**12.**	**Fitness to practice**
	(This competency is about the doctor’s awareness of when his/her own performance, conduct or health, or that of others might put patients at risk and the action taken to protect patients)

Overall there appears to be a paucity of evidence on how postgraduate training in general practice (or lack of) may have a visible and negative impact on the safety of patients – arguably it may be impossible to ever demonstrate clear causation. However, we know that a range of issues connected with the postgraduate training environment may act as proxy indicators of the safety of patient care being compromised unnecessarily and avoidably. For instance, medical educators may be involved in failures of, or inadequate, clinical supervision [15]; or fail to respond appropriately to trainees’ seeking professional guidance [16]; or conduct insufficient joint reviews of the management of complex clinical cases [17]; or provide limited feedback on drug prescribing performance [18]; and may let poorly developed attitudes and behaviour (such as lack of insight) continue unchecked [19]. Other salient issues that are potentially safety-critical include trainees’ possessing different levels of clinical knowledge [20] and an inability to prioritise their clinical workloads and manage time [17]. The quality of the learning environment in which trainees are based may also affect the safety of patient care [21], while doctors-in-training are known to be susceptible to medical errors [6,22].

Given what is known about human error theory in the healthcare workplace [23], and the marked differences in local safety cultures [24] and the reliability of practice systems [5-9], it is inevitable that variation in the quality of training provision exists and that some issues will be inadequately covered or even overlooked completely. If this happens with fundamental training topics which are considered to be safety-critical then there is a likelihood that the risk of patients being harmed and other quality of care issues arising could potentially increase.

Evidence is accumulating from healthcare safety and improvement initiatives that routine adherence to the adoption and use of checklist reminders can improve the overall reliability with which care processes and tasks are undertaken and so potentially mitigate future risks [25-28]. Against this background, we aimed to identify and prioritise the most safety-critical issues to be addressed by educational supervisors and trainees in the first 12-weeks of specialty training in the general practice environment – the training period judged to be specifically high risk and beyond which trainees are given greater clinical freedom. In doing this we may help maximize early opportunities to address safety-critical issues proactively via a checklist reminder, which may lead to a reduced risk of patients being unintentionally harmed during and after the training period.

## Methods

We used a six-stage mixed methods approach to develop and content validate the checklist for educational supervisors (Table 3).

**Table 3:** Summary of study methods: sequential timeline and individual stages

**Stage**	**Month/Year**	**Participants & Location**	**Purpose**	**Methods**	**Outcome**
**1.**	April 2010	72 GP Educational Supervisors (South East Region of Scotland, UK)	· To identify safety-critical educational issues to be covered in early GPST	· Three 90-min facilitated workshops	· Generation of 12 Flipchart sheets of qualitative data on issues perceived to be safety-critical during the first 12 weeks of GPST in the general practice environment
				· Group work and feedback	
**2.**	June 2010	2 Educational Researchers (PB & JM, Glasgow, UK)	· To code, categorise and theme qualitative data	· Thematic analysis	· Generation of 18 Domains and 67 Items perceived to be safety-critical educational issues to be covered during early GPST
			· To identify from key published literature on patient safety and primary care those issues with direct relevance to the GP training environment		
**3.**	July to October 2010	6 Educators (5 GPST Programme Directors and 1 General Practice Manager, Glasgow, UK)	· To validate and refine existing Domains and Items and identify issues not covered.	· Modified Delphi group method (three rounds) utilising list of Domains and Items using electronic mail	· Further refinement: 15 Domains and 55 Items
			· To explore potential uses of the information collected		· Agreement on development of a Checklist (for educational supervisors) and s Self-Assessment Tool (for GP trainees)
**4.**	February 2011	11 GPST Course Organisers and 1 Training Practice Manager	· To further validate and refine Domains and Items deemed to be essential for the first 12 weeks of GPST and identify issues not covered	· Pre-workshop reading and reflection	· Further refinement of Checklist/Self-Assessment Tool content: 14 Domains and 47 Items
			· To explore issues around the acceptability and feasibility of the proposed Checklist and Self-Assessment Tool	· A single 4-h facilitated workshop	
				· Group work and feedback	
**5.**	June to August 2011	9 GP Trainees	· To validate and refine Domains and Items deemed to be essential for the first 12 weeks of GPST and identify issues not covered	· A mix of workshop discussion (n = 3, 2 h) and four 30 min telephone interviews	· No essential safety-critical issues raised for this stage of training
**6.**	August 2011	24 GP Educational Supervisors, Renfrewshire, UK	· Final validation of checklist Domains and Items	· Completion of a Content Validity Index (CVI)	· Final agreement on a Checklist comprising 14 Domains and 47 related Items.

### Preliminary theme and item content generation

As a first step we held three 90-minute workshops in April 2010 with a total of 72 experienced GP educational supervisors (known previously as ‘trainers’) at the south east of Scotland annual GP training conference. They were asked to prioritise their perceptions of the six most critical threats to patient safety in the GP specialty training (GPST) environment (beyond specific clinical topics). In each workshop 4 or 5 groups of supervisors reflected on this task for around 45 minutes, provided feedback on their findings (via a flip chart) and engaged in open discussion about how this information could be of value in the training environment. The workshop facilitator (PB) made contemporaneous field notes and retained all the flip chart data with consent from participants. The authors then independently reviewed each chart and coded the text before generating a preliminary list of safety-critical themes and items (specific educational and training issues) of relevance to early GPST. Differences in theme formulation and item nesting were jointly negotiated until agreement was reached.

We then identified and examined a small number of published reviews and grey literature on the key threats to patient safety in primary care internationally [5-9,29-33]. The information collated was evaluated by the authors and a small number of safety-critical themes and related items of specific relevance to GPST were highlighted. We augmented our aforementioned list with these data to generate an updated preliminary checklist consisting of 15 safety-critical themes and 67 related items.

We invited and recruited a convenience sample of six highly experienced GP specialty training programme directors and course organisers based in the west of Scotland to participate in a modified-Delphi process (three rounds by electronic mail) to gain consensus on the checklist domains and items that were judged to be ‘essential’ to the first 12-weeks of specialty training in the general practice setting. This further iterative refinement resulted in the checklist being reduced to 14 safety-critical themes with 55 related items.

### Further input and validation from educational supervisors and specialty trainees

We invited a random sample of 25 West of Scotland regional GP educational supervisors by electronic mail to reflect on the relevance of each of the 55 items to the first 12-weeks of training in the GP environment and then attend and participate in a follow-up 3-hours workshop to further review and endorse the checklist content. A total of 21/25 (84%) completed both tasks leading to further refinement and updating of content themes (n = 14) and items (n = 47). Discussion with this group also resulted in the proposed final checklist being adapted as a self-assessment tool for specialty trainees.

To further triangulate our findings, we sent the checklist content for review to nine volunteer specialty trainees who had recently completed their initial training period in the GP environment. We held a follow-up 2-hour workshop (n = 3) and telephone discussions (n = 6) with the trainees who further validated the content. Additional issues were raised by them which were judged by the authors to be relevant to the quality of the learning environment trainees were based in, rather than being deemed essential safety-critical issues in the first 12-weeks of training.

### Content validity index (CVI) exercise

Finally, a further 24 GP educational supervisors from a single geographical district in the west of Scotland undertook a CVI exercise. The relevance of each retained checklist item was assessed by asking the supervisors to rate them using a validated [34] 4-point scale: (*1 = not relevant, 2 = somewhat relevant, 3-quite relevant and 4 = very relevant*). Twenty out of 24 supervisors were required to endorse each item by assigning a rating of at least 3 out of 4, to establish content validity beyond the 0.05 level of significance. This was determined to provide sufficient evidence for inclusion of each item as part of the final checklist. Supervisors were also asked to identify any missing items that they deemed important for inclusion, but after reflective consideration and open discussions no more items were added, deleted or amended.

### Ethical review

The study proposal was pre-screened by the west of Scotland research ethics committee but judged to be a service development not requiring ethical approval.

## Results

The study identified 14 safety-critical domains and 47 related items that were judged to be essential components of specialty training in general practice to be addressed by educational supervisors and trainees in the first 12-week period of training in the general practice setting. Each item was also aligned with one or more of the 12 RCGP curriculum competencies to assist and guide supervisors in delivering the curriculum and collecting supporting evidence (Table 4). All checklist items were endorsed by a minimum of 20 of 24 GP educational supervisors who rated each item ≥3 on the 4-point scale. The overall content validity index ratio for the tool was 0.98 (Table 5).

**Table 4:** Validated safety checklist themes and related items mapped against 12 RCGP curriculum competencies

**Checklist Theme and Item**	**RCGP Curriculum Competency No. [Table**2**]**
**PRESCRIBING SAFELY**	
1. Knowledge of high risk medications (e.g. NSAIDs & Warfarin, Methotrexate)	[5, 6]
2. Controlled Drugs (e.g. knowledge of storage, dose adjustment, prescription format)	[5, 12]
3. Awareness of Health Board/Formulary Prescribing Guidance	[9]
4. Knowledge of practice repeat prescribing system	[7]
5. Risks associated with signing repeat & special requests without consulting records	[5, 6]
6. Monitoring drug side-effects (e.g. Myalgia with Statins)	[5, 6]
**DEALING WITH MEDICAL EMERGENCY**	
7. Ensuring Adequate Emergency Treatment Knowledge/Confirmation of CPR Knowledge & Skills (in past 12 months)	[5]
8. Surgery Emergency Bag/Tray & Equipment	[5]
9. Contents of Doctors’ Emergency Bag/Case (where appropriate)	[5]
10. Awareness of Emergency Contacts (e.g. Ambulance, Police, Social Work…)	[5]
**SPECIFIC CLINICAL MANAGEMENT**	
11. Recognising & Acting on Red Flags for Serious Illness (e.g. patient needs immediate admission or urgent outpatient referral)	[3]
**DEALING EFFECTIVELY WITH RESULTS OF INVESTIGATION REQUESTS**	
12. Need to follow-up & act on results and hospital letters	[12]
13. Knowledge of practice system for results handling	[7]
**PATIENT REFERRALS**	
14. Identifying the need for referral (i.e. recognition of condition requiring further investigation and/or treatment)	[3]
15. Referral system (e.g. how and when to refer ‘urgently’ and ‘routinely’	[7, 9]
16. Clinical appropriateness of referral (e.g. ensure correct clinical priority and correct specialty)	[9]
17. Quality of acute referral letter (e.g. past medical history, medication status, social circumstances)	[7]
**EFFECTIVE & SAFE COMMUNICATION**	
18. Knowledge of internal communication processes within the practice (e.g. e-mail, message systems, practice meetings…)	[7]
19. How to liaise with and understand the roles of team members: who, purpose, how, where, when?	[8]
20. Safe communication with patients and relatives (e.g. consultations, phone calls and letters).	[4]
**CONSULTING SAFELY**	
21. How to safety-net (face-to-face)	[1]
22. How to safety-net (when providing telephone advice)	[1]
23. Awareness of guidelines for use of Chaperones	[11, 12]
**ENSURING CONFIDENTIALITY**	
24. Avoiding breaches of confidentiality	[11]
25. Appropriate disclosure of medical and personal information	[11]
**AWARENESS OF THE IMPLICATIONS OF POOR RECORD KEEPING**	
26. Failing to keep records	[12]
27. Failing to keep accurate records	[12]
28. Failing to confirm patient identify	[12]
29. Failing to document all patient contacts	[12]
30. Knowledge of related legal issues	[12]
**RAISING AWARENESS OF PERSONAL RESPONSIBILITY**	
31. Awareness of professional accountability	[12]
32. Recognising the limits of own clinical competence	[12]
33. How and when to seek help	[12]
34. Personal organisation and effectiveness	[12]
**DEALING WITH CHILD PROTECTION ISSUES**	
35. Recognition of harm and the potential for harm in children	[2]
36. How to liaise with other agencies	[8]
37. Breaching confidentiality	[11, 12]
**ENHANCING PERSONAL SAFETY**	
38. How to access emergency alarms/panic button for personal safety	[12]
39. Dealing with aggressive & violent patients	[12]
40. Ensuring personal safety and security on home visits	[12]
**EMPHASISNG THE IMPORTANCE OF THE LEARNING ENVIRONMENT**	
41. Ensure rapid access to supervisory advice, feedback and support	[10]
42. Raise awareness of practice team contribution and support	[10]
43. Ensure reflective learning recorded in E-Portfolio	[10]
44. Knowledge of clinical audit and significant event analysis	[10]
**SAFE USE OF PRACTICE COMPUTERISED SYSTEMS**	
45. Ensure proficiency in using practice computer system	[7]
46. How to prioritise computer system safety alerts (e.g. Yellow and Red Traffic lights)	[7]
47. The need to avoid common pitfalls (e.g. leaving notes open and writing up the wrong patient)	[7]

**Table 5:** Levels of agreement: GP educational supervisors (n = 24) rating each checklist item (n = 47) ≥3 on the 4-point rating scale and the related content validity index ratio

**Checklist Item No.**	**Number of GP Supervisors in Agreement (n)**	CVI*	**Checklist Item No.**	**Number of GP Supervisors in Agreement (n)**	CVI*
**1**	24	1.00	**25**	23	0.96
**2**	24	1.00	**26**	23	0.96
**3**	22	0.92	**27**	23	0.96
**4**	22	0.92	**28**	24	1.00
**5**	24	1.00	**29**	24	1.00
**6**	24	1.00	**30**	23	0.96
**7**	24	1.00	**31**	23	0.96
**8**	24	1.00	**32**	24	1.00
**9**	24	1.00	**33**	23	0.96
**10**	24	1.00	**34**	23	0.96
**11**	24	1.00	**35**	24	1.00
**12**	24	1.00	**36**	23	0.96
**13**	24	1.00	**37**	24	1.00
**14**	24	1.00	**38**	24	1.00
**15**	24	1.00	**39**	24	1.00
**16**	22	0.92	**40**	24	1.00
**17**	24	1.00	**41**	23	0.96
**18**	24	1.00	**42**	22	0.92
**19**	23	0.96	**43**	22	0.92
**20**	24	1.00	**44**	22	0.92
**21**	24	1.00	**45**	23	0.96
**22**	24	1.00	**46**	23	0.96
**23**	23	0.96	**47**	23	0.96
**24**	23	0.96			

* Mean CVI ratio = 0.98.

## Discussion

We identified and prioritised a whole range of safety-critical issues that were judged to be ‘essential’ as part of the high risk 12-week phase of specialty training in the general practice environment. We demonstrated that the content validity of the checklist tool is adequate, providing the first steps towards validating this approach as a method for educational supervisors to guide and support safety-critical education interventions at this phase of training and potentially in future training periods (Additional file 1). At the suggestion of supervisors, the checklist was also adapted to enable trainees to self-assess safety-related learning needs early in their specialty training and monitor future progress (Additional file 2).

At a fundamental level, the domains and related items generated in this study have the potential to be used by GP Educational Supervisors and General Practice Managers to modernize existing Induction Packs, or help inform the development of new induction processes. More specifically, the tool can be applied by supervisors as a checklist prompt or reminder to ensure that the most safety-critical educational tasks are actually carried out and are done so timeously, efficiently, and without ambiguity. The potential to improve the *reliability* of educational provision in these important areas of specialty training and health care practice should be evident. Completion of the checklist could also be used as supporting evidence to the postgraduate deanery (e.g. during training accreditation visits) that the core patient safety element of the training curriculum is being proactively considered and delivered.

All of these factors are of prime importance because of the potential medico-legal implications for the GP educational supervisor if a trainee is inadequately tutored and supervised and patient safety is subsequently compromised. GP educators need to have mechanisms in place to make an early assessment of a trainee’s competencies and to undertake regular performance reviews and feedback sessions as part of an overall strategy to minimise risks. It should be noted that as trainees’ progress through training they are exposed to a more ‘positive’ and less ‘reductionist’ view of care quality in general practice via engagement with a range of improvement methods and exposure to the Quality & Outcomes Framework as part of routine clinical practice.

Theory suggests that because human memory and attention are imperfect [24], one way to mitigate errors or lapses in the execution of tasks is through the use of procedural checklists to help us perform more reliably. Compliance checklists to improve the safety or reliability of processes are standard practice in high-reliability industries [35,36], most notably in commercial aviation and petro-chemical organisations. In health care checklist use is well documented, particularly in surgical [37], obstetric [38] and intensive care [39] settings where their implementation is associated with improved clinical outcomes, team working and productivity efficiencies.

However, despite these positive reports there is some scepticism over whether the introduction of a checklist on its own is the single greatest factor attributable to improvement success. Bosk et al. [40] suggest that the widespread deployment of a checklist may not be successful without a clear understanding that it is a ‘technical’ solution being introduced to a complex dynamic working environment where pre-existing ‘socio-cultural’ issues may need to be contained or resolved. Clinicians may resist or feel threatened by checklist implementation because they perceive it to impinge on their expertise, interfere with their decision-making or over-simplify the working environment. We also know that there is variation in perceptions of local safety climate within and between general practice teams and that some may unintentionally inflate this measure because of inexperience with, or lack of knowledge of, local safety concerns [41]. Similar variation exists in the strength of team working [42], the maturity of the learning environment [21] and commitment to quality improvement [43]. Attending to these types of socio-cultural issues is, therefore, as equally important as supporting efforts into checklist development and implementation if momentum is to be gained and a successful impact made.

Adaptation of the checklist as a self-assessment tool for trainees was an unexpected study development. Self-assessment is a method of measuring and interpreting one’s own performance and is a well-established educational intervention amongst all health care professions and as part of specialty training [44]. The use of self-assessment tools by specialty trainees to identify learning needs is routine in medicine [45,46], but does not appear to be well developed in terms of highlighting specific educational interventions related to patient safety. The activity takes on a greater significance when the performance focus is related to the identification of learning need associated with safety-critical education and training. Although the limitations of self-assessmentis well established [44], it is still promoted as a valuable educational activity because it can be used as a baseline measure which prompts joint discussion and monitoring of performance over time by the trainee and supervisor.

### Study strengths and limitations

A key strength is that the study idea and subsequent tool development were driven by the identification of a need for a patient safety education intervention by experienced frontline medical educators. We used mixed methods to explore and capture in-depth qualitative feedback and quantitative measures to further confirm validation of tool content which strengthened the rigour of this methodological process. For instance, the final CVI ratio was very high which may be a reflection of the deep consideration of checklist items by a large number of informed educators.

Limitations included the potential for bias through the pragmatic use of convenience samples of volunteer participants. Issues around multi-ethnicity were not raised by our participants but we recognise that in other areas of the UK and beyond, particularly in larger cities, this may be recognised as a potential safety-critical problem. Demographic data on participants was not collected as we judged that this would not necessarily add value to study findings and may hinder recruitment. Our key interest was in tapping into trainers’ knowledge, experiences and perceptions of the safety-critical elements of training.

We only recruited a small number of trainees to validate both the checklist and self-rating scale (which they would use) as it was important to triangulate our findings and get their perspective on safety-critical issues. This was useful in terms of confirming rather than adding to our findings, but exploring these issues with more trainees may have resulted in deeper insights.

Although content validation processes were robust, the tools have yet to be adequately tested and evaluated for evidence of acceptability, feasibility and educational gain. Determining how the successful implementation and wide-spread use of the checklist would directly impact on safety outcomes may not be possible, which means we may need to define proxy measures of success.

## Conclusions

The dissemination and implementation of the checklist and rating scale are proceeding on a national, voluntary basis in Scottish GP specialty training practices with plans in place to evaluate their feasibility and educational impact in the short-term. There is clear potential for patients to be unintentionally and avoidably harmed because of what happens (or does not happen) educationally in the GP training environment. The checklist concept is but one small (albeit untested) intervention to help minimise the related risks to patients and the potential medico-legal consequences for supervisor and trainee alike [47]. The use of a checklist measure to ensure the reliable delivery of ‘essential’ patient safety education has potential relevance for all medical specialties and other clinical professions with similar training arrangements in the UK and internationally.

## Competing interests

The authors declare that there are no competing financial or non-financial interests.

## Authors’ contributions

PB conceived the study idea, acquired funding, led the study design, data collection, analysis and interpretation, and drafted the initial manuscript. JM contributed to study design and data collection, and the content and critical review of the manuscript. MK contributed to study design, and the content and critical review of the manuscript. All authors read and approved the final manuscript.

## Pre-publication history

The pre-publication history for this paper can be accessed here:

http://www.biomedcentral.com/1471-2296/13/62/prepub

## Supplementary Material

Additional file 1 GPST Safety Checklist. Click here for file

Additional file 2 GPST Safety Self-Rating Scale. Click here for file

## References

[B1] Brennan TA Leape LL Laird NM Hebert L Localio AR Lawthers AG Newhouse JP Weiler PC Hiatt HH Incidence of adverse events andnegligence in hospitalized patients. Results of the Harvard MedicalPractice Study N Engl J Med 1991 324 370 376 10.1056/NEJM199102073240604 1987460

[B2] World Health Organization Patient safety research: better knowledge for better care 2009 WHO, Geneva

[B3] Department of Health An organisation with amemory: report of an expert group on learning from adverse events in the NHS 2000 HMSO, London

[B4] Department of Health Doing less harm:improving the safety and quality of care through reporting, analysing and learning from adverse incidents involving NHS patients – Key requirements for healthcare providers 2001 HMSO, London

[B5] Sandars J Esmail A The frequency and nature of medical error in primary care: understanding the diversity across studies Fam Pract 2003 20 3 231 236 10.1093/fampra/cmg301 12738689

[B6] McKay J Bradley N Lough M Bowie P A review of significant events analysed in general medical practice: implications for the quality and safety of patient care BMC Fam Pract 2009 10 61 10.1186/1471-2296-10-61 19723325 PMC2744665

[B7] Elder NC Dovey SM Classification of medical errors and preventable adverse events in primary care: a synthesis of the literature J Fam Pract 2002 51 927 932 12485545

[B8] Makeham MAB Dovey SM County M Kidd MR An international taxonomy for errors in general practice: a pilot study Med J Aust 2002 177 68 72 12098341 10.5694/j.1326-5377.2002.tb04668.x

[B9] Dovey SM Meyers DS Phillips RL A preliminary taxonomy of medical errors in family practice Qual Saf Health Care 2002 11 233e8 12486987 10.1136/qhc.11.3.233 PMC1743626

[B10] Scottish Patient Safety Alliance http://www.patientsafetyalliance.scot.nhs.uk/programme [Accessed 4th January 2012}

[B11] Health Protection Scotland NHS Scotland National HAI Prevalence Survey Final Report. Volume 2 of 2. NHS Scotland National HAI Prevalence Survey Protocol

[B12] National Patient Safety Agency Seven steps to patient safety for primary care 2005 NPSA, London

[B13] World Alliance for Patient Safety WHO Patient safety curriculum guide for medical schoolshttp://www.who.int/patientsafety/activities/technical/who_ps_curriculum.pdf (Accessed 31st August 2011)

[B14] Royal College of General Practitioners GP Curriculum Statements 2011 RCGP, London http://www.rcgp-curriculum.org.uk/rcgp_curriculum_documents.aspx [Accessed 4th January 2012]

[B15] Kilminster SM Jolly BC Effective supervision in clinical practice settings: a literature review Med Educ 2000 34 827 840 10.1046/j.1365-2923.2000.00758.x 11012933

[B16] Bruijn M Busari JO Wolf BHM Quality of clinical supervision as perceived by specialist registrars in a university and district teaching hospital Med Educ 2006 40 1002 1008 10.1111/j.1365-2929.2006.02559.x 16987191

[B17] Kilminster SM Jolly BC Grant J Good supervision: guiding the clinical educator of the 21st century 2000 University of Sheffield, Sheffield Department of Health Publication; European Working Time Directive, 2008

[B18] Dornan T Ashcroft DM Heathfield H Lewis PJ Miles J Taylor D Tully MP Wass V FINAL report: An in depth investigation into causes of prescribing errors by foundation trainees in relation to their medical education - EQUIP study 2009 General Medical Council, UK

[B19] Kennedy TJ Lingard L Baker GR Clinical oversight: conceptualizing the relationship between supervision and safety J Gen Intern Med 2007 22 1080e5 17557190 10.1007/s11606-007-0179-3 PMC2305735

[B20] Van der Vleuten CP Schuwirth LW Assessing professional competence: From methods to programmes Med Educ 2005 39 3 309 317 10.1111/j.1365-2929.2005.02094.x 15733167

[B21] Smith VC Wiener-Ogilvie S Describing the learning climate of general practice training: the learner’s perspective Educ Prim Care 2009 20 6 435 440 20132638 10.1080/14739879.2009.11493831

[B22] Zwart DLM Heddema WS Vermeulen MI van Rensen ELJ Verheij TJM Kalkman CJ Lessons learnt from incidents reported by post graduate trainees in Dutch general practice: A prospective cohor tstudy BMJ Qual Saf 2011 20 857e862 doi:10.1136/bmjqs.2010.045484 10.1136/bmjqs.2010.045484 21602562

[B23] Singh H Thomas EJ Petersen LA Medical errors involving trainees: a study of closed malpractice claims from 5 insurers Arch Intern Med 2007 167 2030e6 17954795 10.1001/archinte.167.19.2030

[B24] Reason JT Vincent CA Understanding adverse events: the human factor Clinical risk management. Enhancing patient safety 2001 2 Blackwell BMJ books, London 9 30

[B25] National Patient Safety Agency WHO Surgical Safety Checklist 2009 NPSA, London

[B26] Shillito J Arfanis K Smith A Checking in healthcare safety: theoretical basis and practical application Int J Health Care Qual Assur 2010 23 699 707 10.1108/09526861011081831 21125965

[B27] Semel ME Resch S Haynes AB Funk LM Bader A Berry WR Weiser TG Gawande AA Adopting a surgical safety checklist could save money and improve the quality of care in U.S. hospitals Health Aff (Millwood) 2010 29 1593 1599 10.1377/hlthaff.2009.0709 20820013 PMC3069616

[B28] Hales BM Pronovost PJ The checklist tool for error management and performance improvement J Crit Care 2006 21 231e5 16990087 10.1016/j.jcrc.2006.06.002

[B29] Makeham M Dovey S Runciman W Methods and Measures used in Primary Care Patient Safety Research: Results of a literature review http://www.who.int/patientsafety/research/methods_measures/makeham_dovey_full.pdf [Accessed 12th December 2011]

[B30] O’Beirne M Sterling P Zwicker K Safety incidents in family medicine BMJ Qual Saf 2011 20 1005 1010 10.1136/bmjqs-2011-000105 21893612

[B31] Jacobs S O’Beirne M Derfiingher LP Errors and adverse events in family medicine: developing and validating a Canadian taxonomy of errors Can Fam Physician 2007 53 271e6 271e270 17872644 PMC1949126

[B32] De Wet C Bowie P A preliminary study to develop and test a global trigger tool to identify undetected error and patient harm in primary care records Postgrad Med J 2009 85 176 180 10.1136/pgmj.2008.075788 19417164

[B33] Hoffmann B Beyer M Rohe J ‘Every error counts’: a web-based incident reporting and learning system for general practice Qual Saf Health Care 2008 17 4 307 312 10.1136/qshc.2006.018440 18678731

[B34] Yaghmaie F Content validity and its estimation J Med Educ 2003 3 1 23 25

[B35] Weick K Kathleen M Managing the Unexpected-Assuring High Performance in an Age of Complexity 2001 Jossey-Bass, San Francisco, CA, USA

[B36] Degani A Wiener E Human Factors of Flight-Deck Checklists: The normal Checklist 1990 NASA, Moffet Field, California

[B37] Haynes AB Weiser TG Berry WR A surgical safety checklist to reduce morbidity and mortality in a global population N Engl J Med 2009 360 491e9 19144931 10.1056/NEJMsa0810119

[B38] Rao K Lucas DN Robinson PN Surgical safety checklists in obstetrics Int J Obstet Anesth 2010 19 235e40 20219348 10.1016/j.ijoa.2009.09.008

[B39] Gawande A The checklist: if something so simple can transform intensive care, what else can it do? New Yorker 2007 10 86e101 18084821

[B40] Bosk CL Dixon-Woods M Goeschel CA Pronovost PJ The art of medicine: Reality check for checklists Lancet 2009 374 444 445 10.1016/S0140-6736(09)61440-9 19681190

[B41] de Wet C Johnson P Mash R McConnachie A Bowie P Measuring perceptions of safety climate in primary care: a cross-sectional study 2010 18 1 135 42 Epub 2010 Sep 22 10.1111/j.1365-2753.2010.01537.x 20860593

[B42] Campbell SM Hann M Hacker J Burns C Oliver D Thapar A Mead M Safran DS Roland MO Identifying predictors of high quality care in English general practice: observational study Br Med J 2001 323 7316 784 787 10.1136/bmj.323.7316.784 11588082 PMC57358

[B43] Apekey TA McSorley G Tilling M Siriwardena AN Room for improvement? Leadership, innovation culture and uptake of quality improvement methods in general practice J Eval Clin Pract 2011 17 2 311 318 10.1111/j.1365-2753.2010.01447.x 20438607

[B44] Colthart I Bagnall G Evans A The effectiveness of self-assessment on the identification of learner needs, learner activity, and impact on clinical practice: BEME Guide no. 10 Med Teach 2008 30 124 145 10.1080/01421590701881699 18464136

[B45] Davis DA Mazmanian PE Fordis M Accuracy of physician self-assessment compared with observed measures of competence: a systematic review JAMA 2006 296 1094 1102 10.1001/jama.296.9.1094 16954489

[B46] Overeem K Faber M Arah O Doctor performance assessment in daily practice: does it help doctors or not? A systematic review Med Educ 2007 41 1039 1049 10.1111/j.1365-2923.2007.02897.x 17973764

[B47] Mackenzie P Anthony S The role of the GP trainer:medico-legal aspects Clin Gov: An Int J 2009 14 1 74 79 10.1108/14777270910933479

