# Supplementary material for: Maximising harm reduction in early specialty training for general practice: validation of a safety checklist
Source: BMC Fam Pract. 2012 Jun 21;13:62. doi: 10.1186/1471-2296-13-62 (PMC3418214; doi:10.1186/1471-2296-13-62)
Supplement: Additional file 1 — GPST Safety Checklist. [file 1471-2296-13-62-S1.doc]

Specialty Training for General Practice

***SAFETY CHECKLIST FOR EDUCATIONAL SUPERVISORS***

**Essential Issues to be Covered During the Initial 12-week Training Period in the General Practice Setting**

**SPECIALTY TRAINEE NAME:**

***Suggested Mapping t**o nMRCGP Competencies

| **A. PRESCRIBING SAFELY** | | ***Competency** | **X** |
| --- | --- | --- | --- |
| **1** | Knowledge of high risk medications (e.g. NSAID & Warfarin, Methotrexate) | 5, 6 |  |
| **2** | Controlled Drugs (e.g. knowledge of storage, dose adjustment, prescription format) | 5, 12 |  |
| **3** | Awareness of Health Board/Formulary Prescribing Guidance | 9 |  |
| **4** | Knowledge of practice repeat prescribing system | 7 |  |
| **5** | Risks associated with signing repeat & special requests without consulting records | 5, 6 |  |
| **6** | Monitoring drug side-effects (e.g. Myalgia with Statins) | 5, 6 |  |
| **COMMENTS** | | | |
| **B. DEALING WITH MEDICAL EMERGENCY** | | ***Competency** | **X** |
| **7** | Ensuring Adequate Emergency Treatment Knowledge/Confirmation of CPR Knowledge & Skills (in past 12 months) | 5 |  |
| **8** | Surgery Emergency Bag/Tray & Equipment | 5 |  |
| **9** | Contents of Doctors’ Emergency Bag/Case (where appropriate) | 5 |  |
| **10** | Awareness of Emergency Contacts (e.g. Ambulance, Police, Social Work…) | 5 |  |
| **COMMENTS** | | | |
| **C. SPECIFIC CLINICAL MANAGEMENT** | | ***Competency** | **X** |
| **11** | Recognising & Acting on Red Flags for Serious Illness (e.g. patient needs immediate admission or urgent outpatient referral) | 3 |  |
| **COMMENTS** | | | |
| **D. DEALING EFFECTIVELY WITH RESULTS OF INVESTIGATION REQUESTS** | | ***Competency** | **X** |
| **12** | Need to follow-up & act on results and hospital letters | 12 |  |
| **13** | knowledge of practice system for results handling | 7 |  |
| **COMMENTS** | | | |
| **E. PATIENT REFERRALS** | | ***Competency** | **X** |
| **14** | Identifying the need for referral (i.e. recognition of condition requiring further investigation and/or treatment) | 3 |  |
| **15** | Referral system (e.g. how and when to refer ‘urgently’ and ‘routinely’ | 7, 9 |  |
| **16** | Clinical appropriateness of referral (e.g. ensure correct clinical priority and correct specialty) | 9 |  |
| **17** | Quality of acute referral letter (e.g. past medical history, medication status, social circumstances) | 7 |  |
| **COMMENTS** | | | |

| **F. EFFECTIVE & SAFE COMMUNICATION** | | ***Competency** | **X** |
| --- | --- | --- | --- |
| **18** | Knowledge of internal communication processes within the practice (e.g. e-mail, message systems, practice meetings…) | 7 |  |
| **19** | How to liaise with and understand the roles of team members: who, purpose, how, where, when? | 8 |  |
| **20** | Safe communication with patients and relatives (e.g. consultations, phone calls and letters). | 4 |  |
| **COMMENTS** | | | |
| **G. CONSULTING SAFELY** | | ***Competency** | **X** |
| **21** | How to safety-net (face-to-face) | 1 |  |
| **22** | How to safety-net (when providing telephone advice) | 1 |  |
| **23** | Awareness of guidelines for use of Chaperones | 11, 12 |  |
| **COMMENTS** | | | |
| **H. ENSURING CONFIDENTIALITY** | | ***Competency** | **X** |
| **24** | Avoiding breaches of confidentiality | 11 |  |
| **25** | Appropriate disclosure of medical and personal information | 11 |  |
| **COMMENTS** | | | |

| **I. AWARENESS OF THE IMPLICATIONS OF POOR RECORD KEEPING** | | ***Competency** | **X** |
| --- | --- | --- | --- |
| **26** | Failing to keep records | 12 |  |
| **27** | Failing to keep accurate records | 12 |  |
| **28** | Failing to confirm patient identify | 12 |  |
| **29** | Failing to document all patient contacts | 12 |  |
| **30** | Knowledge of related legal issues | 12 |  |
| **COMMENTS** | | | |
| **J. RAISING AWARENESS OF PERSONAL RESPONSIBILITY** | | ***Competency** | **X** |
| **31** | Awareness of professional accountability | 12 |  |
| **32** | Recognising the limits of own clinical competence | 12 |  |
| **33** | How and when to seek help | 12 |  |
| **34** | Personal organisation and effectiveness | 12 |  |
| **COMMENTS** | | | |

| **K. DEALING WITH CHILD PROTECTION ISSUES** | | | | ***Competency** | **X** |
| --- | --- | --- | --- | --- | --- |
| **35** | Recognition of harm and the potential for harm in children | | | 2 |  |
| **36** | How to liaise with other agencies | | | 8 |  |
| **37** | Breaching confidentiality | | | 11, 12 |  |
| **COMMENTS** | | | | | |
| **L. ENHANCING PERSONAL SAFETY** | | | | ***Competency** | **X** |
| **38** | | | How to access emergency alarms/panic button for personal safety | 12 |  |
| **39** | | | Dealing with aggressive & violent patients | 12 |  |
| **40** | | | Ensuring personal safety and security on home visits | 12 |  |
| **COMMENTS** | | | | | |
| **M. EMPHASISNG THE IMPORTANCE OF THE LEARNING ENVIRONMENT** | | | | ***Competency** | **X** |
| **41** | | Ensure rapid access to supervisory advice, feedback and support (e.g. with an experienced, contactable and approachable GP)? | | 10 |  |
| **42** | | Raise awareness of practice team contribution and support | | 10 |  |
| **43** | | Ensure reflective learning recorded in E-Portfolio | | 10 |  |
| **44** | | Knowledge of clinical audit and significant event analysis | | 10 |  |
| **COMMENTS** | | | | | |

| **N. SAFE USE OF PRACTICE COMPUTERISED SYSTEMS** | | ***Competency** | **X** |
| --- | --- | --- | --- |
| **45** | Ensure proficiency in using practice computer system | 7 |  |
| **46** | How to prioritise computer system safety alerts (e.g. Yellow and Red Traffic lights) | 7 |  |
| **47** | The need to avoid common pitfalls (e.g. leaving notes open and writing up the wrong patient) | 7 |  |
| **COMMENTS** | | | |

| **FURTHER COMMENTS (where necessary)** | **ACTION PLAN (where necessary)** |
| --- | --- |
|  |  |
