# Supplementary material for: Maximising harm reduction in early specialty training for general practice: validation of a safety checklist
Source: BMC Fam Pract. 2012 Jun 21;13:62. doi: 10.1186/1471-2296-13-62 (PMC3418214; doi:10.1186/1471-2296-13-62)
Supplement: Additional file 2 — GPST Safety Self-Rating Scale. [file 1471-2296-13-62-S2.doc]

Specialty Training for General Practice

**12-WEEK SELF-ASSESSMENT CHECKLIST FOR SPECIALTY TRAINEES**

**To be Completed around 12 weeks into Training Period in General Practice Setting to Assess Levels of Confidence in Safety-Critical Areas**

**Specialty Trainee Name:**

**Level of Confidence Rating Scale (1 to 5): Where 1=Low, 3=Moderate, 5=High**

| **No.** | **Safety-Critical Area** | **Confidence level** | | **Comments** |
| --- | --- | --- | --- | --- |
| **1** | Knowledge of high risk medications (e.g. NSAID & Warfarin, Methotrexate) |  | |  |
| **2** | Controlled Drugs (e.g. knowledge of storage, dose adjustment, prescription format) |  | |  |
| **3** | Awareness of Health Board/Formulary Prescribing Guidance |  | |  |
| **4** | Knowledge of practice repeat prescribing system |  | |  |
| **5** | Risks associated with signing repeat & special requests without consulting records |  | |  |
| **6** | Monitoring drug side-effects (e.g. Myalgia with Statins) |  | |  |
| **7** | Adequate Emergency Treatment/CPR Knowledge & Skills |  | |  |
| **8** | Awareness of Surgery Emergency Bag/Tray & Equipment |  | |  |
| **9** | Contents of Doctors’ Emergency Bag/Case (where appropriate) |  | |  |
| **10** | Awareness of Emergency Contacts (e.g. Ambulance, Police, Social Work…) |  | |  |
| **11** | Ability to Recognise & Act on Red Flags for Serious Illness (e.g. patient needs immediate admission or urgent outpatient referral |  | |  |
| **12** | Ability to follow-up & act on results and hospital letters |  | |  |
| **13** | Your knowledge of practice system for results handling |  | |  |
| **14** | Identifying the need for referral (same as before?) |  | |  |
| **15** | Referral system |  | |  |
| **16** | Clinical appropriateness of referral |  | |  |
| **17** | Quality of acute referral letter |  | |  |
| **18** | Knowledge of internal communication processes within the practice (e.g. e-mail, message systems, practice meetings…) |  | |  |
| **19** | How to liaise with and understand the roles of team members: who, purpose, how, where, when? |  | |  |
| **20** | Ability to communicate safely with patients and relatives (e.g. consultations, phone calls and letters). |  |  | |
| **21** | How to safety-net (face-to-face) |  |  | |
| **22** | How to safety-net (when providing telephone advice) |  |  | |
| **23** | Awareness of guidelines for use of Chaperones |  |  | |
| **24** | Avoiding breaches of confidentiality |  |  | |
| **25** | Appropriate disclosure of medical and personal information |  |  | |
| **26** | Need to keep records |  |  | |
| **27** | Need to keep accurate records |  |  | |
| **28** | Need to confirm patient identify |  |  | |
| **29** | Knowledge of implications of failing to document all patient contacts |  |  | |
| **30** | Knowledge of legal issues related to poor record keeping |  |  | |
| **31** | Awareness of your professional accountability |  |  | |
| **32** | Ability to recognise the limits your own clinical competence |  |  | |
| **33** | Your ability on how and when to seek help |  |  | |
| **34** | Your personal organisation and effectiveness |  |  | |
| **35** | How to recognise harm and the potential for harm in children |  |  | |
| **36** | How to liaise with other agencies about child protection issues |  |  | |
| **37** | How to maintain confidentiality around child protection issues |  |  | |
| **38** | How to access emergency alarms/panic button for personal safety |  |  | |
| **39** | Dealing with aggressive & violent patients |  |  | |
| **40** | Ensuring personal safety and security on home visits |  |  | |
| **41** | How to rapidly access supervisory advice, feedback and support (e.g. with an experienced, contactable and approachable GP)? |  |  | |
| **42** | Awareness of practice team contribution and support |  |  | |
| **43** | Recording reflective learning in E-Portfolio |  |  | |
| **44** | Knowledge of clinical audit and significant event analysis |  |  | |
| **45** | Proficiency in using practice computer system |  |  | |
| **46** | How to prioritise computer system safety alerts (e.g. Yellow and Red Traffic lights) |  |  | |
| **47** | The need to avoid common pitfalls (e.g. leaving notes open and writing up the wrong patient) |  |  | |

| **COMMENTS** | **ACTION PLAN** |
| --- | --- |
|  |  |
